# Residues from Homologous Transmembrane Helices 4 and 10 Are Critical for P-Glycoprotein (ABCB1)-Mediated Drug Transport

**DOI:** 10.3390/cancers15133459

**Published:** 2023-07-01

**Authors:** Hadiar Rahman, Mark J. Ware, Andaleeb Sajid, Sabrina Lusvarghi, Stewart R. Durell, Suresh V. Ambudkar

**Affiliations:** Laboratory of Cell Biology, Center for Cancer Research, National Cancer Institute, National Institutes of Health, Bethesda, MD 20892-4256, USA; hadiar.rahman@nih.gov (H.R.); markjware4@gmail.com (M.J.W.); andaleeb.sajid@nih.gov (A.S.); sabrina.lusvarghi@fda.hhs.gov (S.L.); durells@mail.nih.gov (S.R.D.)

**Keywords:** ABC transporter, chemoresistance, drug transport, multidrug resistance, mutational analysis, molecular dynamics simulations, P-glycoprotein, transmembrane helices, structure and function

## Abstract

**Simple Summary:**

P-glycoprotein (P-gp, ABCB1) is an ATP-binding cassette (ABC) transporter that contributes to the development of multidrug resistance (MDR) in cancer cells. P-gp pumps various amphipathic agents including anticancer drugs out of cells. The elucidation of the P-gp mechanism of drug transport is critical to develop strategies to overcome MDR. We used interdisciplinary approaches including cell biological, biochemical, mutational analysis, and molecular dynamics simulations to understand the conformational changes that occur in the homologous transmembrane helices (TMHs) 4 and 10 from the inward-open to the ATP-bound inward-closed states of P-gp during the transport cycle. We found that after substituting seven residues in either TMH4 or TMH10 with alanine, there was no significant effect on the transport function. However, the TMH4,10-14A mutant with 14 substitutions lost the ability to transport most of the substrates tested, revealing that conformational changes in both TMHs 4 and 10 are critical for P-gp’s transport function.

**Abstract:**

P-glycoprotein (P-gp, ABCB1) transports structurally dissimilar hydrophobic and amphipathic compounds, including anticancer drugs, thus contributing to multidrug-resistant cancer. Cryo-EM structures of human P-gp revealed that TMHs 4 and 10 contribute to the formation of the drug-binding cavity and undergo conformational changes during drug transport. To assess the role of the conformational changes in TMH4 and TMH10 during drug transport, we generated two mutants (TMH4-7A and TMH10-7A), each containing seven alanine substitutions. Analysis of the drug efflux function of these mutants using 15 fluorescent substrates revealed that most of the substrates were transported, indicating that even seven mutations in an individual helix have no significant effect on transport function. We then designed the TMH4,10-14A mutant combining seven mutations in both TMHs 4 and 10. Interestingly, when the TMH4,10-14A mutant was tested with 15 substrates, there was no efflux observed for fourteen. The basal ATPase activity of the TMH4,10-14A mutant, similar to that of the WT protein, was inhibited by zosuquidar but was not stimulated by verapamil or rhodamine 6G. Molecular dynamics simulations indicated that the mutations cause TMHs 4 and 10 to pack tighter to their proximal helices, reducing their independent mobility. In aggregate, our findings demonstrate the critical role of the residues of homologous TMHs 4 and 10 for substrate transport, consistent with conformational changes observed in the structure of P-gp.

## 1. Introduction

The development of multidrug resistance (MDR) is a frequent problem in treating cancer and a critical public health concern. MDR is defined as the resistance of cancer cells to anticancer drugs through multiple different mechanisms [1]. Numerous studies demonstrated that overexpression of some of the ATP-binding cassette (ABC) transporters is involved in the development of MDR to targeted chemotherapy, resulting in poor prognoses in the treatment outcomes [2,3,4,5]. P-glycoprotein (P-gp; ABCB1) is the most studied member of the family of mammalian ABC transporters. This transporter uses the energy from ATP-binding and hydrolysis to pump out many structurally dissimilar anticancer drugs, thus reducing the intracellular concentrations of anticancer drugs necessary to kill cancer cells [6,7,8]. 

P-gp is a single polypeptide chain which consists of four domains: two transmembrane domains (TMDs), each with six transmembrane helices (TMHs), and two nucleotide-binding domains (NBDs). TMD1 and NBD1 comprise the N-terminal half, homologous to the C-terminal half composed of TMD2 and NBD2 [9,10]. The two TMDs create a large drug-binding pocket in the membrane, and NBDs bind and hydrolyze ATP. P-gp shows polyspecificity in binding and transporting a broad range of substrates; thus, extensive effort has been made to understand the molecular basis of the polyspecificity of this transporter. Our previous studies involving several single, double, or triple mutations in P-gp showed that the expression and substrate transport by mutant P-gp were not altered [11]. The structures of human P-gp resolved by cryo-electron microscopy (EM) indicate that when both the substrate Taxol and the third-generation inhibitor zosuquidar’s bound positions are superimposed, forty residues in the binding cavity interact with these important drugs [12]. To characterize the effect of the alteration of the binding pocket on transport function, mutagenesis and biochemical studies were conducted involving tyrosine substitutions of fifteen residues in the drug-binding pocket [13]. The study demonstrated that the mutant P-gp tolerated these tyrosine substitutions, with virtually no alteration in cell surface expression or transport of small to moderate-size substrates, indicating the flexibility of the drug-binding pocket. 

Biochemical characterization, molecular modeling, and structure–activity relationship studies suggest that P-gp comprises a large drug-binding pocket with overlapping sites for different substrates. We proposed that this structural flexibility contributes to the binding of substrates to multiple sites, forming the basis of its polyspecificity. Recent structural data have provided insights into ligand-binding properties of P-gp, showing that substrates only bind in the central cavity; however, inhibitors can bind in both the central cavity and in a smaller adjoining cavity or “vestibule” [14]. This indicates that multiple residues are involved within these drug-binding pockets to accommodate different ligands. Interestingly, the structural evolution of ABC transporters suggests that the P-gp sequence arose likely from gene duplication. DNA sequence analysis revealed that TMD1 and TMD2 are homologous, with a high degree of sequence identity [15]. To further understand the mechanism of polyspecificity and drug transport, we chose the approach to substitute multiple conserved residues in a pair of homologous helices of P-gp with alanine. Previously, using this strategy, mutations were created in homologous helices TMH1 and TMH7 to generate TMH1,7, a mutant P-gp containing six alanine (Ala) substitutions in each helix. The TMH1,7 mutant was able to transport only 3 out of 25 substrates, demonstrating a loss of broad substrate specificity [16]. A subsequent study with TMHs 6 and 12 generated a 14A mutant containing seven Ala mutations in each helix. The TMH6,12-14A mutant failed to transport most of the tested substrates. However, it surprisingly gained the ability to import certain substrates, including rhodamine123 and flutax-1 [17]. Both studies provided a framework and foundation to study another important pair of homologous helices, TMH4 and TMH10, to determine their role in the drug transport function.

The recent cryo-EM structure of human P-gp indicates that both TMHs 4 and 10 may play an important role in ligand binding in the central cavity by forming a gate-like structure at the cytoplasmic end of the cavity. Based on the structure and in silico analysis, it appears that the helix-breaker amino acids (proline and glycine) in TMHs 4 and 10 play a critical role, blocking the release of the substrate to the cytoplasm in the substrate-bound conformation [12,18,19]. In contrast, structures of mouse P-gp show that straightening of these helices upon ATP-binding in the outward-facing (or referred to here as inward-closed) conformation seems to facilitate the release of the substrate from the cells [20,21,22,23]. 

Although structural studies provide insights into the conformational changes in TMHs 4 and 10 in the inward-open and ATP-bound inward-closed states of human P-gp and other ABC transporters [24,25,26], no biochemical or functional data concerning the role of residues in TMH4 and TMH10 are available. To address this, we substituted multiple conserved residues of TMHs 4 and 10 with alanine and expressed the mutant in HeLa cells using the BacMam baculovirus transient expression system [27]. We found that the transport function of the mutants containing seven Ala substitutions either in TMH4 or 10 alone is almost the same as that of the wild-type (WT) P-gp. However, when the same seven residues are mutated together in both TMHs 4 and 10, the TMH4,10-14A mutant loses the ability to transport most tested substrates. These data, consistent with the flexible nature of the TMDs, indicate that multiple mutations in a single TMH (either TMH4 or TMH10) are well tolerated. Additional data including molecular dynamics (MD) simulations suggest that residues in TMHs 4 and 10 function cooperatively to generate conformational changes necessary for the translocation of substrate drugs out of cells. Thus, our findings provide a functional correlation with the observed structural changes in TMHs 4 and 10 in the inward-open and -closed states of P-gp.

## 2. Materials and Methods

### 2.1. Chemicals and Antibodies

The fluorescent substrates BODIPY (BD)-verapamil, BODIPY-3-propionyl ethylenediamine hydrochloride (BD-EDA), BD-prazosin, and tetramethylrhodamine ethyl ester perchlorate (TMRE) were purchased from Serateh Biotech (Eugene, OR, USA). Other fluorescent compounds including 3-quinolinium 6-(dimethylamino)-2-[4-[4-(dimethylamino)-phenyl]-1,3-butadienyl]- 1-ethyl perchlorate (LDS-751), SYTO13, rhodamine 123 (Rh 123), calcein-acetoxymethyl (Cal-AM), Rhod-2-AM, rhodamine-6G (R6G), X-Rhod-1-AM, and rhodamine B hexyl ester (R6) were purchased from Invitrogen/Thermo Fisher (Carlsbad, CA, USA). Flutax-1 was purchased from Tocris Biosciences (Minneapolis, MN, USA). Cyclosporine A was purchased from the Alexis Corporation (Lausen, Switzerland). [N-ε(4-nitro-benzofurazan-7-yl)-D-Lys(8)]-cyclosporine A (NBD-CsA) was generously provided by Drs Anika Hartz and Bjoern Bauer, University of Kentucky (Lexington, KY, USA). All remaining chemicals were obtained from Sigma-Aldrich (St. Louis, MO, USA). The P-gp-specific monoclonal antibody C219 was provided by Fujirebio Diagnostic Inc. (Malvern, PA, USA), the MRK16 antibody was purchased from Kyowa Medex Company (Tokyo, Japan), and UIC2 antibody was purified from hybridoma cells as described in [28]. FITC-labeled anti-mouse secondary antibody IgG2a κ isotype was obtained from BD Biosciences (San Jose, CA, USA).

### 2.2. Cell Line and Culture Conditions

HeLa-S3 cells (CCL-2.2) were purchased from the American Type Culture Collection (ATCC) and grown in Dulbecco’s modified Eagle’s Medium (Gibco, Thermo Fisher Scientific) supplemented with 10% Fetal Bovine Serum, 5 mM L-glutamine, 100 units/mL penicillin, and 100 μg/mL streptomycin at 37 °C in 5% CO_2_ as described previously [17].

### 2.3. BacMam Baculovirus Transduction of HeLa-S3 Cells and Cell Surface Expression of the Mutants

HeLa-S3 cells were transduced with BacMam baculovirus containing WT or mutant P-gp, as described previously [17,27], except the mutants’ virus-to-cell ratio was adjusted with the WT to have a similar level of cell surface expression. Transduced cells were incubated for 22–24 h. After 24 h, cells were harvested, and 300,000 cells were incubated for 1 h with the human P-gp-specific MRK16 antibody (1 μg/100,000 cells) in IMDM medium containing 5% FBS. These cells were washed and incubated with FITC-labeled secondary antibody for 30 min and then washed with ice-cold IMDM medium. Cell surface expression of P-gp was then analyzed using a FACS Canto II (BD Biosciences, Franklin lakes, NJ, USA), and data were analyzed using FlowJo v10 software (Tree Star Inc., Ashland, OR, USA). To determine the conformation of mutant P-gp at the cell surface, the conformation-sensitive UIC2 antibody reactivity assay was used as described previously [11,28,29,30,31].

### 2.4. Transport of Fluorescent Substrates

The transport function of WT and mutant P-gp was determined using flow cytometry as described previously [13]. Approximately 300,000 cells were incubated with various fluorescent substrates: calcein-AM (0.5 µM) for 10 min; or BD-CsA (0.5 µM), BD-EDA (0.5 µM), BD-prazosin (0.5 µM), BD-verapamil (0.5 µM), daunorubicin (4 µM), flutax-1 (5 µM), DiOC2 (0.5 µM), NBD-CsA (0.5 µM), LDS-751 (0.5 µM), R6G (1 µM), Rh123, (1.3 µM), dihydrorhodamine 123 (1.3 µM), R6 (1 µM), SYTO 13 (0.5 µM), TMRE (1 µM), and X-Rhod-1 AM (0.5 µM) for 45 min. Following the incubation, cells were washed with cold IMDM. Then, 300 μL of PBS containing 0.1% bovine serum albumin (BSA) was added to each tube, followed by determination of efflux of substrates by measuring cell-associated fluorescence using flow cytometry (FACS Canto II, BD Biosciences, Franklin lakes, NJ, USA). The data were analyzed using FlowJo v10 software (Tree Star Inc., Ashland, OR, USA). 

### 2.5. Cytotoxicity Assays

We performed cytotoxicity assays with HeLa-S3 cells expressing WT P-gp or mutant P-gp. Cells transduced with either WT or mutant P-gp BacMam baculovirus were incubated for 24 h and plated in white opaque 96-well plates. Concentration ranges of 0–500 nM of paclitaxel were used, followed by incubation for 48 h at 37 °C. The Cell Titer Glo reagent (Promega, Madison, WI, USA) kit was used, and the percent cell survival was calculated as described earlier [13]. Data were analyzed using GraphPad Prism 9.0 software to calculate IC_50_ values. Three independent experiments were performed. 

### 2.6. Preparation of Membrane Vesicles of High Five Insect Cells

Recombinant baculovirus carrying WT or mutant P-gp with a 6X His-tag and TEV-cleavage site at the C-terminal end was used to infect High Five insect cells as described before [32,33]. Total membranes were prepared as described in [34] with some modifications. The aliquots were snap-frozen using dry ice and stored at −80 °C. Total protein was quantified by the Amido black protein estimation method as previously described [35], using bovine serum albumin fraction V as the standard. 

### 2.7. SDS-PAGE and Western Blotting

Total lysates from HeLa-S3 cells expressing WT or mutant P-gp were prepared as described before [16,32,34], with minor modifications. Cells were lysed by sonication and freeze–thaw cycles in a buffer containing 10 mM Tris-Cl pH 8.0, 0.1% Triton X-100, 10 mM MgSO_4_, 2 mM CaCl_2_, 1% aprotinin, 1 mM AEBSF, 2 mM DTT, and 20 μg/mL nuclease. Cell lysate samples (lysate of 60,000 cells per lane) were resolved using a 7% NuPAGE Tris-Acetate 1.5 mm gel for electrophoresis at a constant voltage of 150V for 1 h. Western blotting was performed at a constant current of 400 milli Amp for 1 h. For blocking, the membrane was soaked in 20% nonfat milk in PBST (PBS containing 0.05% Tween-20) for 40 min. After removing the blocking solution, the membrane was incubated overnight with C219 primary antibody (1:1000 dilution) for P-gp [36] and GAPDH primary antibody (1:10,000 dilution) prepared in 5% milk + PBST. The membrane was washed three times for 10 min each with PBST, followed by incubation with a secondary antibody HRP-conjugated goat anti-mouse IgG (1:5000 dilution) for 1 h with shaking, prepared in 5% milk + PBST. The membrane was washed three times with PBST for 5 min each. The blots were developed using an ECL Western blotting detection kit (GE Healthcare, Pittsburgh, PA, USA) and scanned using a Bio-Rad ChemiDoc touch imager. The quantification of the ECL signals was performed by ImageJ (NIH open-source software, Version 1.53t).

To determine P-gp expression levels in High Five insect cells, membrane vesicles (10 μg protein/lane) with WT and mutant P-gp were resolved by 7% Tris-acetate SDS-PAGE and stained with colloidal blue for 1 h, followed by washing overnight. Gels were scanned using a Bio-Rad ChemiDoc touch imager. To prepare a Western blot using the insect cell total membrane vesicles, a similar procedure was used as above and High Five insect membrane vesicles (0.5 μg protein/lane) were loaded on the gel. To detect P-gp levels, we used the P-gp-specific monoclonal C219 antibody (1:2000 dilution) and a secondary antibody (HRP-conjugated goat anti-mouse IgG (1:5000 dilution)). Image J (NIH open-source software) was used to quantify the protein. All the experiments were repeated at least three times, and values represent mean ± SD. 

### 2.8. ATPase Activity

An ATPase assay was performed as described previously [32,36,37]. Briefly, to conduct the ATPase assay, assay buffer containing 50 mM MES-Tris pH 6.8, 50 mM KCl, 10 mM MgCl_2_, 5 mM NaN_3_, 1 mM EGTA, 1 mM ouabain, and 2 mM DTT was prepared and kept refrigerated at 4 °C. Total membrane vesicles (10 μg protein per 100 μL reaction volume) were incubated in the presence or absence of 0.3 mM sodium orthovanadate. The basal activity was measured in the presence of DMSO control, and drug-modulated activity was measured in the presence of 50 μM verapamil. The reaction mixture was acclimated and kept at 37 °C for 5 min. The reaction was initiated by adding 5 mM ATP and continued for 20 min at 37 °C. The reaction was stopped by the addition of 2.5% SDS after 20 min incubation at 37 °C. Using the colorimetric method as described in [36,37], the amount of inorganic phosphate liberated from the reaction was quantified. The vanadate-sensitive ATPase activities were calculated, and data were plotted with GraphPad Prism software (V9). 

### 2.9. Molecular Modeling and Electrostatic Surface Potential

Protein structural graphics and molecular modeling were performed using PyMOL (Version 2.0, Schrodinger open-source software) to study the interactions of TMH4-TMH6 and TMH10-TMH12 using cryo-EM structures of P-gp (PDB- 6QEX and 6C0V) [12,19]. A 4Å cutoff PyMOL script was used to determine the residues’ interactions with the bound ligand. Models of the WT and TMH4,10-14A mutant versions of the protein were generated from the cryo-EM structure of human P-gp in the inward-open conformation (PDB: 6QEX). The bound Taxol molecule was removed, and the 14 Ala substitutions were performed manually in the coordinates to form the mutant structure. The structures were then manually embedded in a membrane bilayer of POPC lipids. These were then used to calculate the electrostatic potential fields with the PBEQ module of the Charmm-46b1 software suite [38]. For modeling, the WT P-gp structure from the Protein Data Bank (PDB: 6QEX) in the apo version was embedded in a POPC bilayer membrane. The mutant protein was derived from the same structure except for the 14 mutations in TMH4,10-14A, which were manually implemented in the coordinate file. The atomic radii and electronic charges were obtained from the CHARMM36m forcefield [39]. A dielectric constant of 1.0 was used for the protein and membrane and a constant of 80.0 was used for the explicit solvent. The solvent probe radius was 1.4 Å, the ion exclusion radius was 2.0 Å, and the temperature was 300 K. To accentuate the fields, an ionic strength of only 10 mM was used. The protein/membrane system was centered in a square grid of 200 Å per side, with a resolution of 0.5 Å per unit grid length.

### 2.10. Molecular Dynamics (MD) Simulations

For molecular dynamics simulations, the two membrane-embedded models described above were further solvated with TIPS3P water molecules with the VMD (version 1.9.3) software package [40]. This included adding Na^+^ and Cl^−^ ions to neutralize the systems and provide an ion strength of 150 mM. MD simulations were conducted with NAMD 2.13b2 software [41] and the CHARMM36 all-atom forcefield [42]. Periodic boundary conditions were used for a rectangular cell of starting dimensions 118.0 × 115.0 × 168.0 Å. Regular electrostatic and van der Waals forces were calculated with a CUTOFF and SWITCHDIST of 12.0 and 8.0 Å, respectively. The temperature and pressure were maintained at 310.0 °K and 1 ATM with the Langevin and Langevin piston methods, respectively. The only constraints were the lengths of the covalent bonds involving hydrogen, which were kept at their equilibrium values to allow for a 2.0 fs time-step. Trajectories were evolved for a minimum of 1400 ns each and then analyzed with the Motion Tree 1.2 software [43] sampling every 100 ps, to obtain the protein fluctuations. 

### 2.11. Statistical Analysis

Statistical analyses were performed with GraphPad-Prism software version 9.0 (GraphPad Software LLC., Boston, MA, USA). The values in the results represent mean values (mean ± standard deviation, SD) of at least three independent experiments.

## 3. Results

### 3.1. Rationale for Studying Homologous Transmembrane Helices TMH4 and TMH10 and the Selection of Residues to Generate Mutants

**Figure 1:**
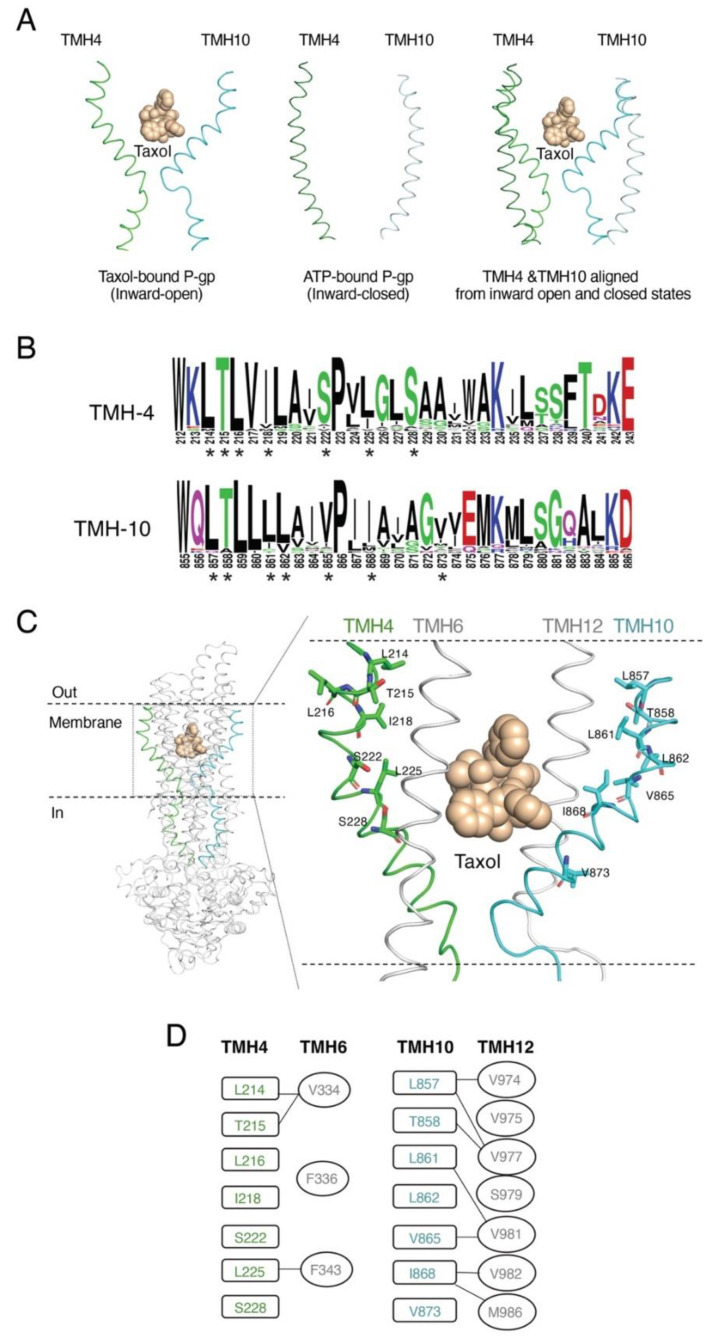
Rationale for selecting residues in TMHs 4 and 10 of P-gp. (**A**) Cartoon representation of TMH4 and TMH10 of human P-gp in two atomic structures. On the left, TMH4 and TMH10 are in an inward-open conformation (PDB ID: 6QEX) with Taxol in the center of the binding pocket [12]. In the middle, both helices are in the ATP-bound inward-closed conformation (PDB: 6C0V) [19]. On the right, superimposition of TMHs 4 and 10 from inward-open (green and cyan ribbon) with inward-closed (light green and light cyan) helices. (**B**) Sequence logo generated with the TMH4 and TMH10 sequence of P-gp from various species using the open-source software Web logo (https://weblogo.berkeley.edu/logo.cgi, accessed on 12 January 2023) The height of the residues in the logo reflects the degree of conservation. Asterisks (*) below the sequence logo indicate the conserved residues selected for mutational analysis. (**C**) Model of the atomic structure of P-gp based on the cryo-EM structure (PDB ID: 6QEX) with Taxol bound. In the cartoon representation of the whole P-gp molecule, TMH4 and TMH10 are highlighted in green and cyan, respectively. The enlarged area shows the residues of TMH 4 and TMH 10 selected for substitution with Ala, shown as green and cyan sticks, respectively. Both TMHs 6 and 12 are shown to indicate their proximity to TMHs 4 and 10. (**D**) Interactions of the TMH4 and TMH10 residues (in boxes) within 4Å of the residues (in ovals) from TMH6 and TMH12 are indicated by connecting lines.

The recent inward-open cryo-EM structure of human P-gp (PDB ID: 6QEX) in complex with Taxol revealed that both TMHs 4 and 10 undergo significant conformational changes in the drug-binding region [12]. In this Taxol-bound structure, both TMHs 4 and 10 have a helical bend and form a gate that opens to the cytoplasm (Figure 1A, left). On the other hand, the inward-closed ATP-bound P-gp structure (PDB ID: 6C0V) indicates that TMHs 4 and 10 are in a linear conformation, and the helical bend is absent (Figure 1A, middle). When both states were superimposed, it became clear that the helical conformation of TMHs 4 and 10 changes dramatically in the inward-closed conformation (Figure 1A, right). This helix bent-to-straight transition may be necessary for the ligand to bind and transport substrates by a peristaltic mechanism [12,14]. These structural patterns suggest the important role of these two helices (TMHs 4 and 10) in the transport function. 

To address the role of residues of TMHs 4 and 10, we generated multiple mutations in both TMHs. First, we chose residues that are highly conserved across species. These conserved residues were identified based on multiple sequence alignments using *ABCB1* sequences from 25 species, ranging from *Caenorhabditis elegans* (worm) to zebrafish, tuna fish, chicken, human, and several other mammalian species. The sequence logo in Figure 1B was generated by aligning the extracted gene sequences, which indicates the most conserved and thus critical residues. Besides the conservation of the residues, we also considered two additional criteria to select residues. The selected residues were preferably located within 4Å of the bound Taxol in the drug-binding pocket in the Taxol-bound structure (Figure 1C). Third, in silico analysis of TMH4, TMH6, TMH10, and TMH12 in both inward-open (PDB ID: 6QEX) and inward-closed (PDB ID: 6C0V) conformations of P-gp indicate the proximity and the interactions of the residues of these helices. MD simulation data suggest that conformational changes in TMH4, TMH6, TMH10, and TMH12 are required for substrate binding and translocation of the substrate through a peristaltic mechanism [14,44]. Using this information, we substituted seven conserved residues in TMH4 (L214, T215, L216, I218, S222, L225, S228) and seven conserved residues in TMH10 (L857, T858, L861, L862, V865, I868, V873) to generate TMH4-7A and TMH10-7A mutants. The selected residues in TMH4 and TMH10 are within 4Å of some of the residues of TMH6 and TMH12 (Figure 1D), which were previously found to be important for substrate transport and change of direction of the transport by P-gp [17].

### 3.2. Both TMH4-7A and TMH10-7A Mutants Have a Similar Level of Expression as WT P-gp

**Figure 2:**
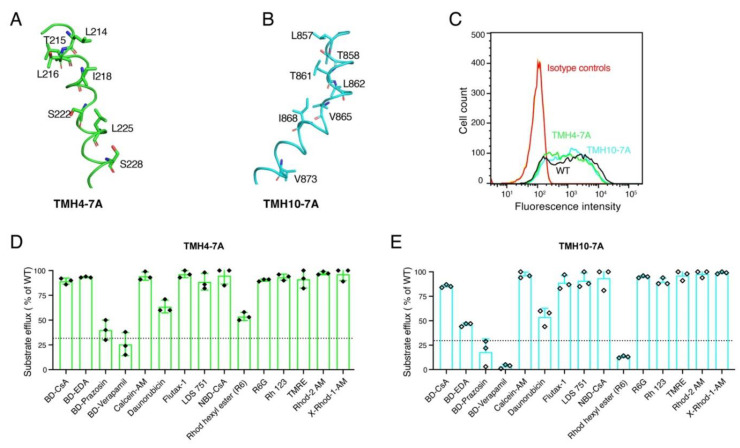
TMH4-7A and TMH10-7A mutants are expressed on the cell surface of HeLa cells and exhibit transport of most substrates, similar to WT P-gp. Cartoon representations of the TMH4 (**A**) and TMH10 (**B**) in green and cyan, respectively. Labeled residues shown as stick models were mutated to Ala to generate TMH4-7A and TMH10-7A mutant P-gps. HeLa-S3 cells were transduced with WT, TMH4-7A, and TMH10-7A mutant P-gp using the BacMam baculovirus system. After 24 h of incubation at 37 °C, cells were harvested and stained with human P-gp-specific monoclonal antibody MRK-16. (**C**) Cell surface expression data from flow-cytometric analysis. MRK-16 staining of cells with WT, TMH4-7A, and TMH10 mutant P-gp are indicated by black, green, and cyan curves, respectively. Isotype controls are in blue. The transport function of (**D**) TMH4-7A mutant P-gp and (**E**) TMH10-7A mutant P-gp for 15 tested fluorescent substrates. The transport efficacy of the mutants was calculated, and error bars indicate the SD of three independent experiments. The dotted line indicates the threshold (<30% compared to efflux by WT) taken as the lowest detectable level or no transport.

Seven Ala substitutions either in TMH4 or TMH10 could possibly alter the cell surface expression and function of the mutant P-gp [11]. To assess the possible effect of seven Ala substitutions in both TMHs 4 and 10 (shown in Figure 2A,B) on the expression of P-gp, we measured cell surface expression. HeLa cells transduced with the WT P-gp served as a positive control. To determine the expression of the TMH4-7A and TMH10-7A mutants, transduced HeLa cells were stained with the human P-gp-specific monoclonal antibody MRK16 as described in the Materials and Methods section. Our results show that the TMH4-7A and TMH10-7A mutant P-gps have an expression level the same as that of WT (Figure 2C), suggesting that seven Ala substitutions did not change the overall surface expression of the mutant P-gps.

### 3.3. Seven Alanine Substitutions in TMHs 4 and 10 Do Not Have Any Significant Effect on the Transport Function

To characterize the transport function of the TMH4-7A and TMH10-7A mutant P-gps, we chose 15 fluorescent substrates that are structurally and functionally diverse amphipathic compounds. These substrates are either intrinsically fluorescent, such as daunorubicin and Rh123, or conjugated with a fluorescent moiety, such as BODIPY (BD) including BD-EDA, BD-prazosin, and BD-verapamil. The substrates R6, Rh123, R6G, Rhod-2 AM, TMRE, and X-Rhod-1 AM are rhodamine-based compounds. Transport assays were performed with HeLa cells expressing WT or mutant P-gps as described in the Materials and Methods section. Using flow cytometry, the fluorescence of compounds retained in the cells was quantified as a measure of substrate retained in the cells. Less fluorescence correlates with efflux, as the substrates are pumped out of cells. Untransduced HeLa cells with undetectable levels of P-gp exhibited the maximal level of fluorescence, which was taken as control (0% efflux). Based on transport efficiency, efflux of substrates within a range of 0–30% compared to the WT level was considered as essentially no transport, while efflux ranges of 30–75% and >75% of WT are considered partial and full transport, respectively. Analysis of the transport data for the TMH4-7A mutant revealed that most of the tested substrates were fully transported. Two substrates (daunorubicin and R6) were partially transported, and two others (BD-verapamil and BD-prazosin) were not transported (Figure 2D). Interestingly, the TMH10-7A mutant P-gp demonstrated a transport profile the same as that of TMH4-7A. While most of the tested substrates were transported by TMH10-7A, two (BD-EDA and daunorubicin) were partially effluxed and three others were not transported (Figure 2E). These data demonstrate that mutations in individual helices TMH 4 or 10 do not significantly affect the transport function of P-gp. 

### 3.4. The Expression and Overall Conformation of the TMH4,10-14A Mutant Is the Same as WT P-gp

**Figure 3:**
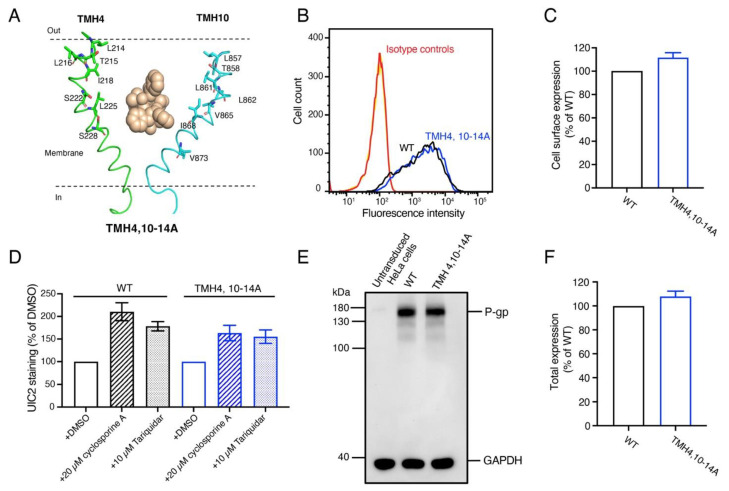
The TMH4,10-14A mutant is expressed in HeLa cells with overall conformation very similar to WT P-gp. (**A**) Cartoon representation of TMH4 (green) and TMH10 (cyan) with Taxol bound in the center of the binding pocket (pdb.6QEX). Residues shown as stick models were mutated to Ala to generate the TMH4,10-14A mutant. (**B**) HeLa-S3 cells were transduced with WT, TMH4,10-14A mutant BacMam baculovirus, stained with the human P-gp-specific monoclonal antibody MRK-16, and analyzed using flow cytometry as described in the Materials and Methods section. Cells expressing WT and TMH4,10-14A mutant P-gp stained with MRK-16 monoclonal antibody are indicated with black and red curves. (**C**) Expression of WT P-gp is taken as 100%, and the relative expression of TMH4,10-14A mutant P-gp was calculated. Three to five independent replicates were quantified, and error bars show SD. (**D**) The overall conformation of the TMH4,10-14A mutant is the same as WT protein. The conformation-sensitive monoclonal antibody UIC2 reactivity assay was performed in the presence of DMSO (solvent), a substrate cyclosporine A (10 µM), or an inhibitor tariquidar (10 µM) as described previously [3,4,5,6,7]. (**E**) Western blot of lysates of HeLa cells transduced with BacMam baculovirus expressing WT and TMH4,10-14A mutant P-gp using the C219 antibody. The lysate of 60,000 cells expressing untransduced cells (lane 1), WT (lane 2), and TMH4,10-14A mutant P-gp (lane 3) were loaded. GAPDH expression was used as a loading control. The PageRuler prestained protein ladder from Thermo Fisher Scientific (Waltham, MA, USA) was used. All these experiments were performed in triplicate. (**F**) Bar graph representing the quantification of the P-gp levels (in panel D) with error bar showing the SD of three independent experiments. The uncropped blots and the molecular weight marker for Figure 3E are shown in Figures S4 and S5.

The above findings suggest that as many as seven mutations in a single helix, either TMH 4 or TMH10, are well tolerated. We then generated a mutant, TMH4,10-14A, with the Ala substitutions of the same residues in both TMHs 4 and 10 (Figure 3A). To check whether 14 alanine substitutions affect the expression levels of the mutant P-gp, HeLa cells expressing TMH4,10-14A and WT P-gp were stained with MRK-16 antibody. The MRK-16 staining demonstrates that the TMH4,10-14A mutant P-gp has a similar cell surface expression level as WT P-gp (Figure 3B,C). To determine whether there was any change in the overall conformation of the TMH4,10-14A mutant at the cell surface, the conformation-sensitive UIC2 reactivity assay was used. The binding of UIC2 to P-gp is increased by pretreatment with cyclosporine A or tariquidar [11,28,29,30]. As shown in Figure 3D, the UIC2 reactivity to the mutant P-gp either in the presence of cyclosporine A or tariquidar is the same as that of the WT transporter, indicating the similar overall conformation. 

Western blot analysis of the lysates of HeLa cells expressing WT and TMH4,10-14A given in Figure 3E,F indicates the total cellular level of mutant P-gp was the same as WT P-gp. These data demonstrate that 14 mutations in TMH4,10-14A do not seem to affect the expression or overall conformation of this mutant. 

### 3.5. TMH4,10-14A Mutant Failed to Transport All the Tested Substrates

**Figure 4:**
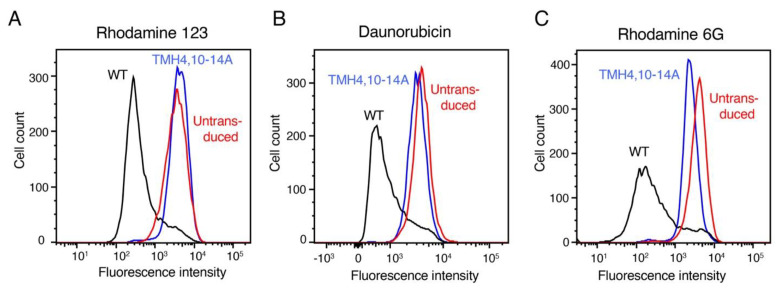
Determination of transport function of the TMH4,10-14A mutant P-gp using selected fluorescent substrates. Hela-S3 cells were used to transduce with TMH4,10-14A mutant and WT P-gp. HeLa cells without transduction served as control. Using flow cytometry, the transport of the fluorescent substrates was measured as described in Materials and Methods. Two substrates, rh-123 (**A**) and daunorubicin (**B**), are not effluxed (10–30% compared to WT) by TMH4,10-14A mutant P-gp. The R6G substrate is partially (30–70% compared to WT) effluxed (**C**). The fluorescence intensity of WT P-gp is shown in black, that of the TMH4,10-14A mutant in red, and untransduced cells as blue curves in all the histograms.

**Figure 5:**
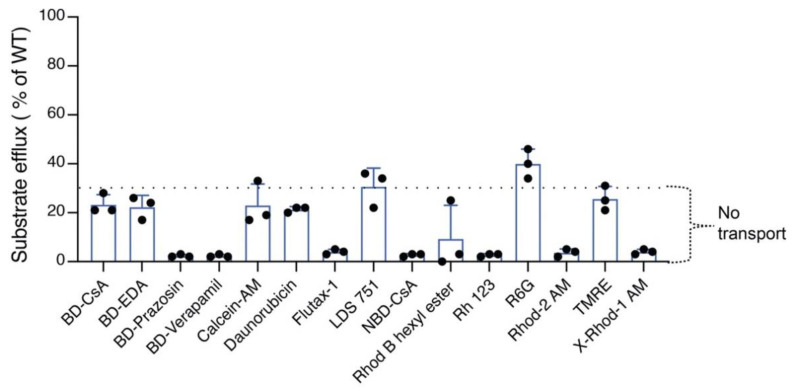
TMH4,10-14A mutant P-gp lost the ability to efflux most tested substrates. Bar graph represents the efflux of 15 substrates by TMH4,10-14A mutant P-gp. The dotted line indicates the threshold (<30% compared to WT) for the lowest level or no transport. Data represent the mean ± SD of at least three independent experiments.

Using the same 15 fluorescent substrates tested with the TMH4-7A and TMH10-7A mutants, we characterized the transport function of the TMH4,10-14A mutant P-gp. In Figure 4, representative histograms indicate the efflux of three of the 15 substrates (rh 123, daunorubicin, and R6G). A decrease in fluorescence intensity is correlated with increased efflux. The three substrates shown are all effluxed by WT P-gp, but the TMH4,10-14A mutant failed to mediate the transport of rh 123 and daunorubicin. As shown in Figure 4C, R6G was partially (~40–45% compared to WT) effluxed by the mutant. Figure 5 shows the efflux of all 15 tested fluorescent substrates by the TMH4,10-14A mutant P-gp. As shown in the bar graph in Figure 5, only R6G was partially transported (43 ± 3%) by the TMH4,10-14A mutant. Remarkably, the mutant could not mediate transport of any of the other 14 substrates tested. 

### 3.6. Although the TMH4,10-14A Mutant Exhibits Basal ATPase Activity the Same as That of WT P-gp, Substrates Fail to Stimulate It

It is well established that the transport function of P-gp requires ATP hydrolysis and substrates, and that inhibitors modulate this activity. Certain substrates such as verapamil stimulate the ATPase activity, whereas inhibitors including zosuquidar and tariquidar inhibit it [18].

**Figure 6:**
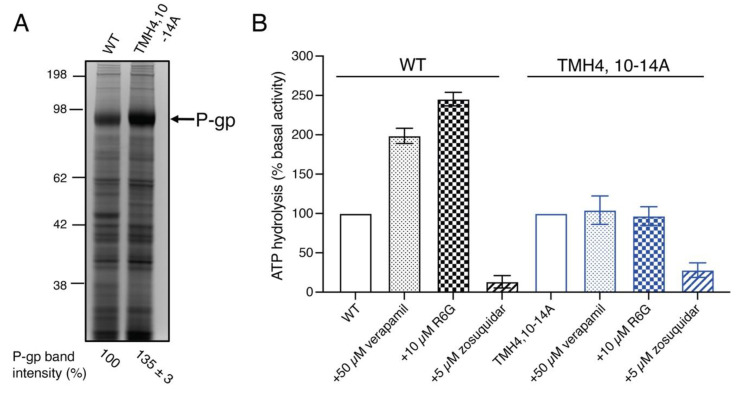
Zosuquidar inhibits the ATPase activity of TMH4,10-14A mutant P-gp, but substrates verapamil and rhodamine 6G fail to stimulate it. Membrane vesicles containing TMH4,10-14A mutant and WT P-gp were prepared from High Five insect cells as described in the Materials and Methods section. (**A**) Both TMH4,10-14A mutant and WT P-gp are expressed to the same level in insect cells. Membrane vesicles (15 μg of protein per lane) were used for SDS-PAGE on 7% Tris-acetate gel, and the gel was stained using Coomassie blue and scanned using a ChemiDoc imager (Bio-Rad). The P-gp protein band was quantified using Image J software. Values at the bottom of the lanes represent mean ± SD from three to five independent experiments. (**B**) ATP hydrolysis by membrane vesicles expressing WT or TMH4,10-14A mutant P-gp was measured in the presence and absence of 0.3 mM sodium orthovanadate, 50 µM verapamil, 10 µM R6G, and 5 µM zosuquidar as described in the Materials and Methods section. Bars show the percent of basal ATPase activity. The vanadate-sensitive basal activity of WT and TMH4,10-14A mutant P-gp was 36.0 ± 3.6 and 34.7 ± 5.0 nanomoles P_i_/min/mg protein and this was taken as 100% [2]. At least three independent experiments were carried out, and each data point represents the mean ± SD. The bar graph with SD was generated using GraphPad Prism 9.0 software. The original protein-stained gel for Figure 6A is shown in Figure S6.

To measure the ATPase activity, we prepared membrane vesicles of WT and TMH4,10-14A mutant P-gp-expressing High Five insect cells. The level of TMH4,10-14A mutant and WT P-gp expressed in insect cell membrane vesicles was compared by performing SDS-PAGE and quantifying stained P-gp bands as described in the Materials and Methods section. We observed that the TMH4,10-14A mutant P-gp had a slightly higher (35 ± 3%) level of expression compared to the WT (Figure 6A), which was considered to normalize the ATPase activity of the mutant. The basal ATPase activity of the TMH4,10-14A mutant P-gp with DMSO solvent alone is similar (34.7 ± 5 nmol P_i_/min/mg protein) to that of WT P-gp (36 ± 3.6 nmol P_i_/mg protein/min) (Figure 6B). To test whether substrates would stimulate the ATPase activity of TMH4,10-14A and WT, we used 50 μM verapamil and 10 µM R6G. Both verapamil and R6G stimulated the ATPase activity of the WT protein, but failed to stimulate the activity of the TMH4,10-14A mutant. We also tested the effect of zosuquidar, an inhibitor of P-gp, and found that at 5 µM concentration, zosuquidar inhibited the basal ATPase activity of both the TMH4,10-14A mutant and WT P-gp (Figure 6B). These data demonstrate that 14 mutations in TMH4,10-14A do not seem to affect the basal ATP activity or its inhibition by an inhibitor, and substrates fail to stimulate activity, consistent with the loss of efflux function of this mutant. 

### 3.7. Mutations in TMH4,10-14A Do Not Significantly Affect the Electrostatic Surface Potential in the Drug-Binding Cavity

As electrostatic interactions can play an important role in drug-binding affinity, we investigated the effect of the 14 mutations on the electrostatic surface potential in the transmembrane cavity. As this can be affected by the low-dielectric medium of the membrane core, it was included in the calculations. Panels A and B of Figure S1 show the results for the WT and TMH4,10-14A mutant P-gps, and panel C shows the difference between the mutant and the WT by subtracting the surface potential of the WT from that of the mutant. The central forms represent the overall shape of the cavity, color-coded for electrostatic potential (blue = positive, white = neutral, and red = negative). As can be seen, the magnitude of the change in potential is relatively small. This is consistent with the fact that 10 mutations are from nonpolar hydrophobic residues to nonpolar alanine, and the remaining 4 are from only polar-neutral to alanine (no change in formal charge). Thus, electrostatic change does not seem to be a significant factor in the loss of drug transport by the mutant. It should be noted, however, that this analysis does not consider possible changes in the cavity shape resulting from mutation-related rearrangements of the TMHs.

### 3.8. MD Simulation of TMHs 4 and 10 of the TMH4,10-14A Mutant and WT P-gp

**Figure 7:**
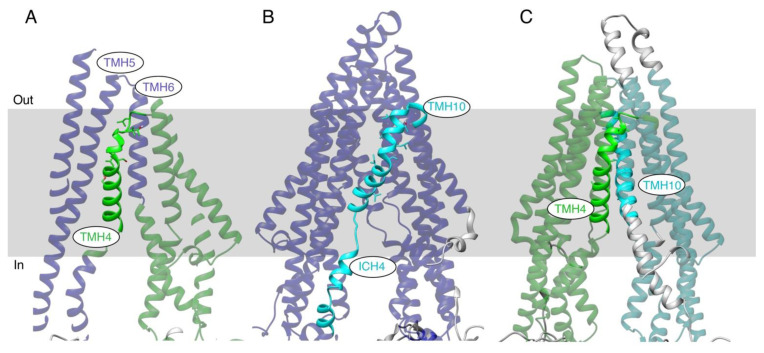
MD simulations of the WT and TMH4,10-14A mutant P-gps. Selected nodes of hierarchical Motion Tree analyses highlight distinct patterns of fluctuations in the helical transmembrane region. Within each panel, the dual colors distinguish the two “rigid” domains of the protein that move independently. Domains containing TMH4 are colored green, TMH10 cyan, and the remaining domains purple. (**A**) WT protein showing the independent movement of TMH4 (bold green) as compared to movement of proximal TMHs 5 and 6 (transparent purple). (**B**) WT protein showing independent movement of TMH10 and ICH4 (bold cyan) from the rest of the TM region. (**C**) TMH4,10-14A mutant protein showing independent, hinge-like motion of the two halves of the TM domain (green and cyan). Helices are shown as ribbons, and the approximate location of the membrane is indicated by a grey rectangle.

The effects of the 14 Ala mutations on the structure of the transporter were investigated by in silico MD simulations. Starting from the inside-open conformation (PDB ID: 6QEX), WT and mutant TMH 4,10-14A versions of the protein were evolved for a total of 1400 ns each. The resultant trajectories were then analyzed with the Motion Tree software program (version 1.2), which provides a hierarchical breakdown of the fluctuations among “rigid-body” subdomains [43]. The Motion Trees of the mutant and WT proteins are given in Figure S2 panels A and B. The main differences between the WT and mutant are illustrated in Figure 7, which shows the results following a 100 ns equilibration phase (i.e., from 100 to 1400 ns). Figure 7A illustrates a Motion Tree node from the WT protein that divides the TMHs into two subdomains (colored blue and green). The maximum fluctuation among the domains at this node is 3.0 Å. Interestingly, TMH4, colored solid green, exhibited movement independent from the movement of the proximal TMH5 and TMH6 helices that pack against it (note that the back of the structure in this panel has been omitted for clarity). A similar situation was found for the WT TMH10, as shown in panel B. In this case, the division is even starker, with TMH10 and Intracellular Helix 4 (ICH4) (colored cyan) moving independently from all the other TMHs (shown in violet). The maximum fluctuation at this node is 3.3 Å. Surprisingly, such independent movements of TMH4 and TMH10 are not observed in the TMH4,10-14A mutant protein. Rather, the node displayed in panel C, which has a maximum fluctuation of 2.3 Å, shows that these mutated helices move in concert with the respective halves of the transmembrane domain. Interestingly, such a hinge-like motion is not observed in the trajectory of the WT protein. These results suggest that the seven alanine substitutions in both TMH4 and TMH10 cause them to pack tighter to their proximal helices, and that their greater, independent mobility in the WT protein is required for the transmembrane domain to convert to the outside-open conformation associated with drug efflux.

### 3.9. Omitting Three Alanine Substitutions in TMH 4 in the TMH4,10-14A Mutant Recovers the Transport of a Few Substrates

Characterization of the TMH4,10-14A mutant revealed that it is essentially nonfunctional. To identify the critical residues in TMH4 important for the transport function, we performed in silico analysis of the conserved residues of TMH4 that are involved in drug binding. Based on our analysis, we found that three conserved residues (L216, I218, and S222) might be important in the transport function. We hypothesized that making a new mutant without mutation of these three conserved residues in TMH4 might indicate whether they are critical for the transport function. Using the TMH4,10-14 template, we generated a new mutant (TMH4,10-11A), which lacks mutations of the three residues (L216, I218, and S222) in TMH4 (Figure S3A). Flow cytometry analysis of the cell surface expression of TMH4,10-11A and WT P-gp is to the same level (Figure S3B,C). We tested the transport function of the TMH4,10-11A mutant P-gp using 15 fluorescent substrates used to test other mutants in this study. Our results revealed that two substrates (R6G and TMRE) were transported at approximately the same level as the WT, and four substrates (BD-EDA, BD-prazosin, daunorubicin, and LDS 751) were partially transported (Figure S3D, and the summary of transport of substrates by four mutants is given in Table 1 for comparison). These data indicate that the TMH4,10-11A mutant, which has only four substitutions in TMH4 compared to seven in TMH4,10-14A, exhibits increased efflux of some of the substrates, suggesting that these three residues (L216, I218, and S222) in TMH4 are important for P-gp-mediated transport.

## 4. Discussion

The overexpression of P-gp, ABCG2, and ABCC1 is one of the major impediments to successful chemotherapy for cancer patients. We used interdisciplinary approaches, including cell and molecular biology, biochemistry, as well as in silico studies, to elucidate the mechanism of drug transport by P-gp, which will provide a basis for developing nontoxic and improved therapeutics to overcome the chemoresistance in cancer cells. We used the BacMam baculovirus-HeLa cell expression system to characterize the structure and function of the WT and variants, as this system allows overexpression of P-gp or other ABC transporters for the assessment of intrinsic properties without exposing cells to any anticancer drugs [11,13,16,17,27].

Despite the availability of detailed structures of P-gp revealing significant conformational changes in TMHs 4 and 10 during the transport cycle [12,30], the lack of biochemical analysis of both TMH4 and TMH10 has hindered our ability to understand the role of the residues in these two helices in drug transport. To analyze the involvement of these helices, we mutated seven conserved residues in each of TMHs 4 and 10 and generated two mutants, TMH4-7A and TMH10-7A. When these mutants were transiently expressed in HeLa cells using BacMam baculovirus, they transported most of the substrates tested ranging in molecular weight from 350 to 1300 Daltons (Figure 2). These findings demonstrated that as many as seven mutations in each of TMH4 and 10 are well tolerated, without affecting the expression or function, further supporting the flexible nature of the drug-binding pocket of P-gp [24,45].

We then investigated whether conformational changes in both TMHs are required for the transport function of P-gp by combining the same seven mutations in TMHs 4 and 10 to generate the TMH4,10-14A mutant. This mutant, even with 14 mutations, was expressed at the cell surface of Hela cells to the same level and with the same overall conformation, determined by using conformation-sensitive monoclonal UIC2 antibody [11,28,29,46], as that of the WT protein (Figure 3). Interestingly, we found that the TMH4,10-14A mutant lost the ability to efflux almost all the substrates tested (Figure 4 and Figure 5). Further analysis of the ATPase activity of the mutant showed that although zosuquidar inhibited the basal activity to the same extent as the WT, two tested substrates failed to stimulate it, which is consistent with the loss of transport function. We then performed MD simulations and found that the independent movements of TMHs 4 and 10 observed in the WT were not observed in the TMH4,10-14A mutant protein. Seven Ala substitutions in both TMH4 and TMH10 appeared to cause them to pack tighter to their proximal helices (Figure 7 and Figure S2). In silico studies indicated that the independent movements of TMHs 4 and 10 in the WT protein are apparently critical for the conformational change to the inward-closed state required for drug efflux. 

The transport of 15 different fluorescent substrates by four mutants was determined as described in the Materials and Methods section. The efflux of each substrate by the WT P-gp was taken as 100%. The values (mean ± SD) given in the table are from three or more independent experiments and depict % efflux compared to WT. Efflux above 70% indicates full transport, 30–70% partial transport, whereas <30% is considered insignificant or no transport. ND = not detected (see the legend to Figure 2A for additional details). 

Our mutational analysis, experimental data, and in silico studies are in agreement with previous molecular simulations, which indicated that TMH4/TMH6 and TMH10/TMH12 form two portals that allow the access of hydrophobic molecules directly from the inner leaflet of the membrane [44]. The movement of these four helices appears to block the portal, presumably to prevent the drug from entering the cytoplasm from the inner leaflet of the membrane [3,19]. We propose that the elucidation of the high-resolution structure of the TMH4,10-14A mutant will provide insight into the altered conformational changes that result in loss of the function of P-gp. Furthermore, this study demonstrates the utility of this novel approach to assess the effect of a group of mutations made to a pair of homologous TMHs to unravel the mechanism of drug transport by P-gp and possibly other ABC drug transporters [25,26].

## 5. Conclusions

In summary, to correlate the major conformational changes in TMHs 4 and 10 during the transport cycle with the function of P-gp, we carried out a mutational analysis and found that substitution of seven residues with Ala in TMH4 or TMH10 alone has no significant effect on the expression or transport function of P-gp, indicating the flexible nature of these helices. On the other hand, when seven mutations in both helices were combined in the TMH4,10-14A mutant, it lost the ability to transport most of the drugs. Additional biochemical and MD simulation data provide further support for the cell-based drug transport data indicating that the conformational changes in both homologous TMHs 4 and 10 are critical for drug transport mediated by P-gp. 

## Figures and Tables

**Table 1:** Summary of the substrate efflux activities of TMH4 and TMH10 mutants.

Substrates	TMH4,10-14A	TMH4,10-11A	TMH4-7A	TMH10-7A
BD-CsA	33 ± 3	3 ± 0.6	89 ± 3	85 ± 2
BD-EDA	22 ± 5	66 ± 6	93 ± 67	46 ± 2
BD-Prazosin	2 ± 0.6	54 ± 4	40 ± 10	18 ± 13
BD-Verapamil	ND	3 ± 0.6	26 ± 12	3 ± 2
Calcein-AM	23 ± 9	58 ± 9	94 ± 4	97 ± 3
Daunorubicin	21 ± 1	34 ± 2	64 ± 6	54 ± 9
Flutax-1	ND	4 ± 2	98 ± 4	92 ± 7
LDS 751	31 ± 8	43 ± 6	89 ± 8	91 ± 8
NBD-CsA	3 ± 1	4 ± 1	95 ± 9	94 ± 11
R6	13 ± 11	6 ± 2	54 ± 6	13 ± 1
R6G	43 ± 3	78 ± 3	90 ± 1	95 ± 1
Rh123	3 ± 0.58	28 ± 5	93 ± 3	90 ± 3
Rhod-2 AM	ND	5 ± 2	96 ± 6	96 ± 5
TMRE	26 ± 5	85 ± 1	100 ± 0	98 ± 3
X-Rhod-1 AM	4 ± 1	4 ± 1	96 ± 6	99 ± 1

## Data Availability

The data can be shared upon request.

## Supplementary Materials

The following supporting information can be downloaded at https://www.mdpi.com/article/10.3390/cancers15133459/s1, Figure S1: electrostatic surface representation of TMH4,10-14A mutant and WT P-gp, Figure S2: hierarchical Motion Trees for WT-P-gp (6QEX-apo) and TMH4,10-14A mutant P-gp, Figure S3: TMH4,10-11A mutant P-gp, which lacks alanine substitution of three conserved residues from TMH4, mediates transport of few substrates, Figure S4: original blot for Figure 3E in the main article, Figure S5: original blot with the molecular weight marker for Figure 3E in the main article, Figure S6: original protein stain for Figure 6A in the main article.
