# Supplementary material for: Residues from Homologous Transmembrane Helices 4 and 10 Are Critical for P-Glycoprotein (ABCB1)-Mediated Drug Transport
Source: Cancers (Basel). 2023 Jul 1;15(13):3459. doi: 10.3390/cancers15133459 (PMC10340712; doi:10.3390/cancers15133459)
Supplement: Supplementary file 1 [file cancers-15-03459-s001.zip › cancers-2420293-supplementary.pdf]

Supplementary information

Residues from homologous transmembrane helices 4 and 10 are critical for P glycoprotein (ABCB1)-mediated drug transport

Hadiar Rahman, Mark Ware, Andaleeb Sajid, Sabrina Lusvarghi, Stewart R. Durell, and Suresh V. Ambudkar*

Laboratory of Cell Biology, Center for Cancer Research, National Cancer Institute, National Institutes of Health, Bethesda, MD 20892-4256, USA

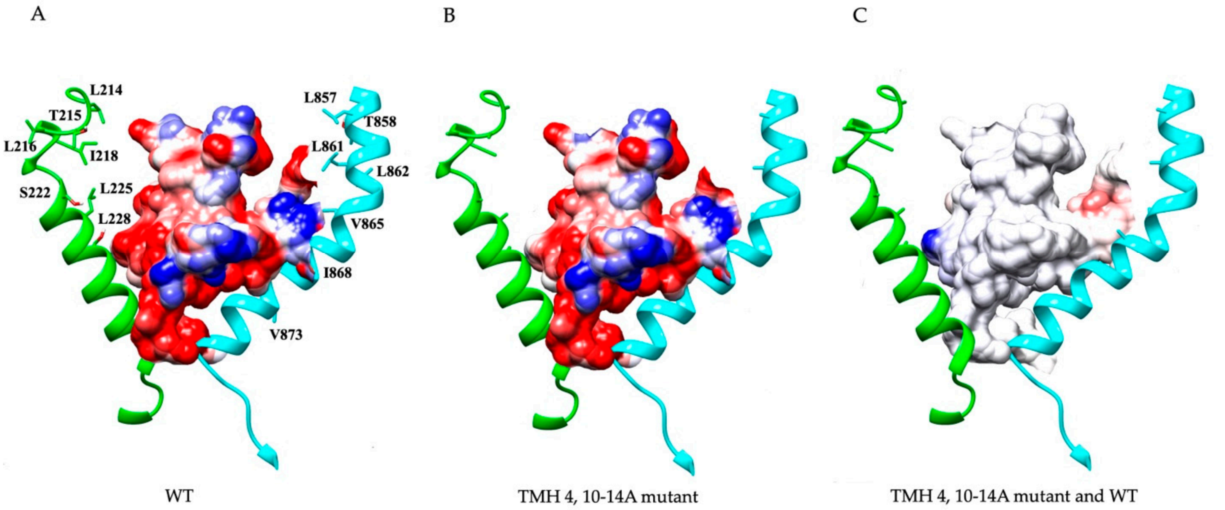

Figure S1. Electrostatic surface representation of TMH4,10-14A mutant and WT P-gp. The atomic structure of human P-gp (PDB: 6QEX) was used to analyze the surface potential of wild-type (WT) and TMH4,10-14A mutant P-gp. The TMH4,10-14A mutant P-gp was generated using the molecular modeling software Chimera (UCSF) and the structure was used to calculate the surface potential. A low dielectric medium of the membrane was used, and the possible effect of the medium is included in the calculation. Panels A and B show the results for the wildtype and TMH4,10-14A mutant forms of the protein, and panel C shows the difference (mutant – wildtype). In WT-apo and TMH4,10-14A-apo, TMH4 and TMH10 are highlighted in green and cyan, respectively. The results of the calculations are shown as color-coded for electrostatic potential. Color code: Blue, positive; red, negative, while indicating neutral.

A

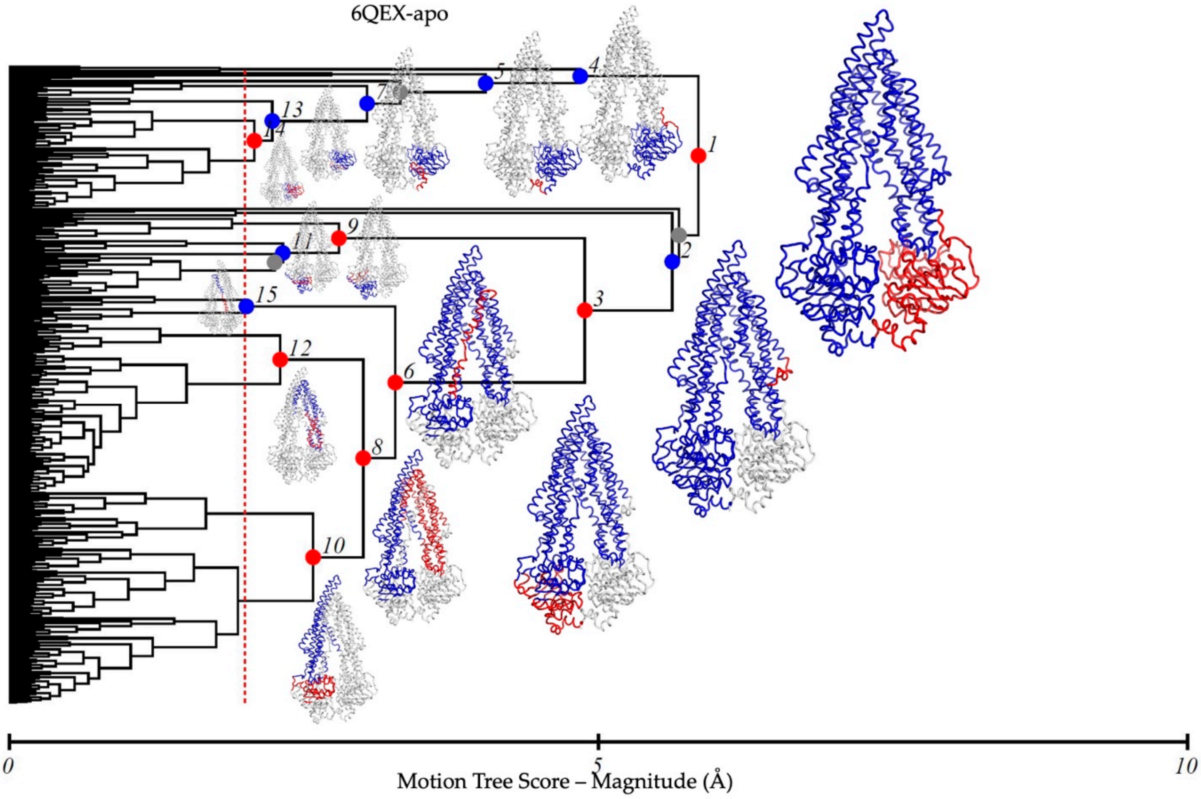
6QEX-apo
1
2
3
4
5
6
7
8
9
10
11
12
13
14
15
0
5
10
Motion Tree Score – Magnitude (Å)

B

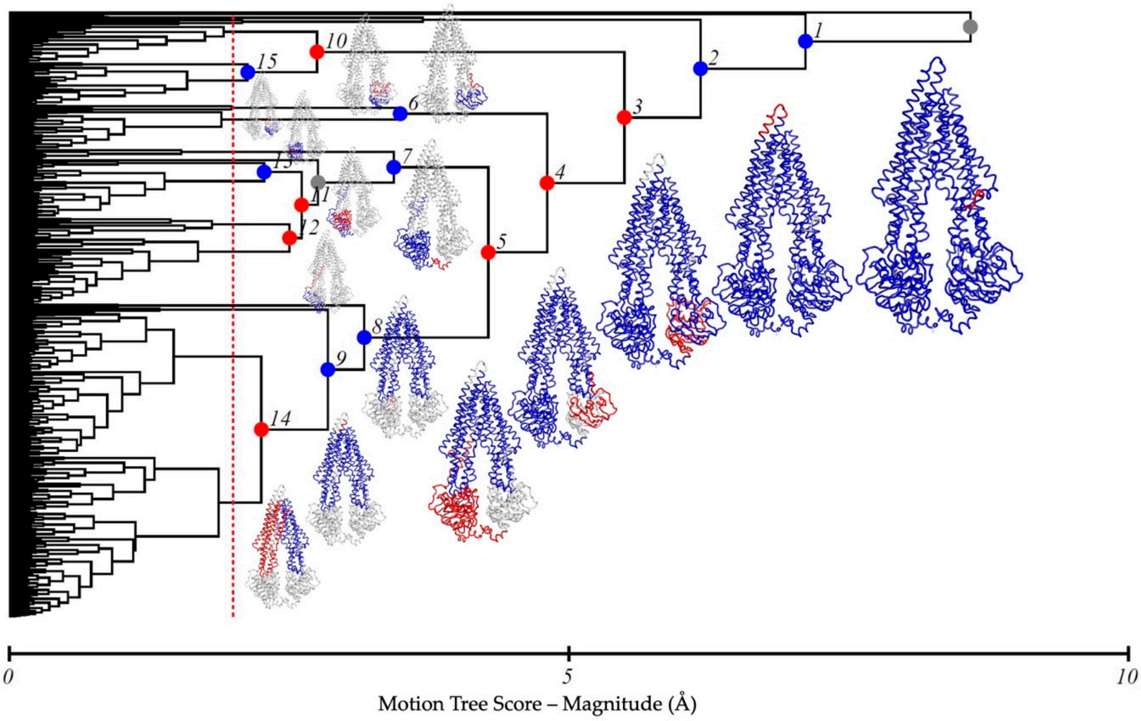
6QEX-apo-TM4,10-14A
1
2
3
4
5
6
7
8
9
10
11
12
13
14
15
0
5
10
Motion Tree Score – Magnitude (Å)

Figure S2. Hierarchical Motion Trees for WT-P-gp (6QEX-apo) and TMH4,10-14A mutant P-gp. The analyses are from 100 to 1400 ns sections of the respective trajectories, sampled every 100 ps. At each node of the dendrograms, the two independently moving, “rigid” domains of the proteins are colored blue and red. The remaining parts are grey. The X-axis represents the magnitude of the fluctuations. Panels A) and B) are the full dendrograms of the WT and TMH4,10-14A mutant proteins.

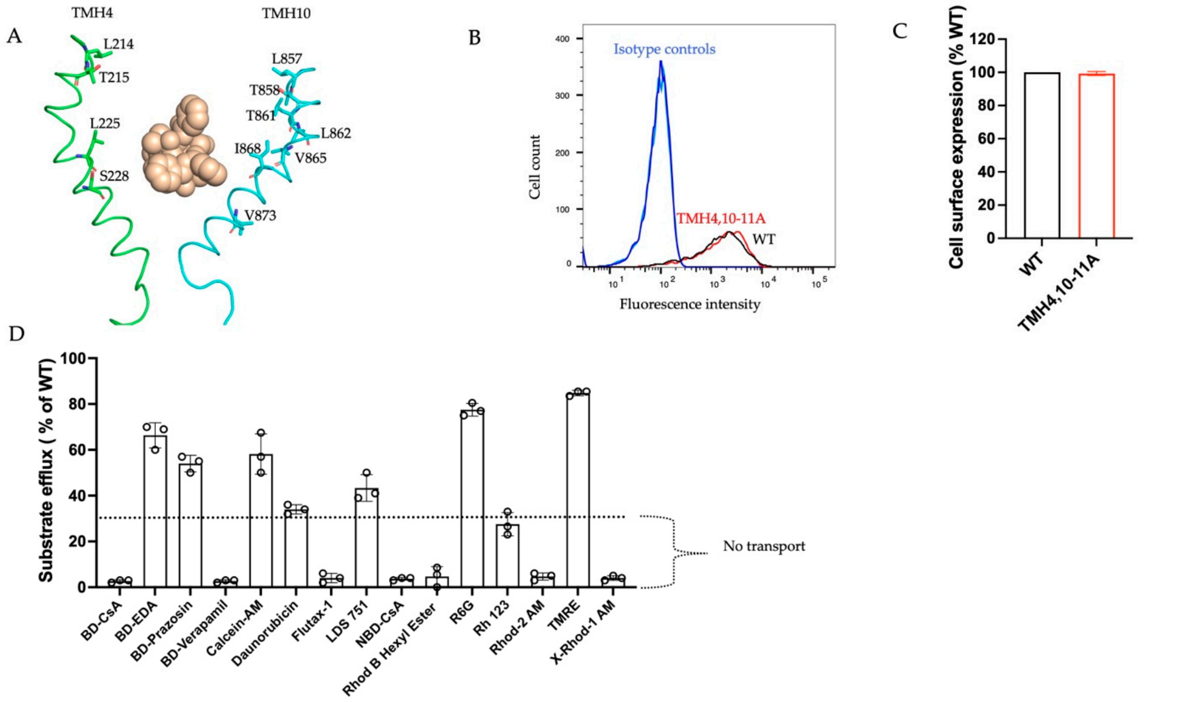

Figure S3. TMH4,10-11A mutant P-gp, which lacks alanine substitution of three conserved residues from TMH4 mediates transport of few substrates. (A) Ribbon representation of TMH4 (green) and TMH10 (cyan) with Taxol at the center of the drug binding cavity in the model of P-gp structure (pdb.6QEX). Four residues from TMH4 (sticks in green) and seven from TMH10 (sticks in cyan) were substituted with ala TMH4,10-11A. (B) Cell surface expression of TMH4,10-11A mutant is similar to WT P-gp. The flow-cytometric analysis of MRK-16 staining. (C) The quantification of the cell surface expression in (B). The WT P-gp level was taken as 100%. Three to five independent replicates were quantified, and error bars show SD. (D) The efflux activity of the TMH4,10-11A with 15 fluorescent substrates.

Figure S4. Original Images for Blots and Gels for the main article.

Figure 3E main blot

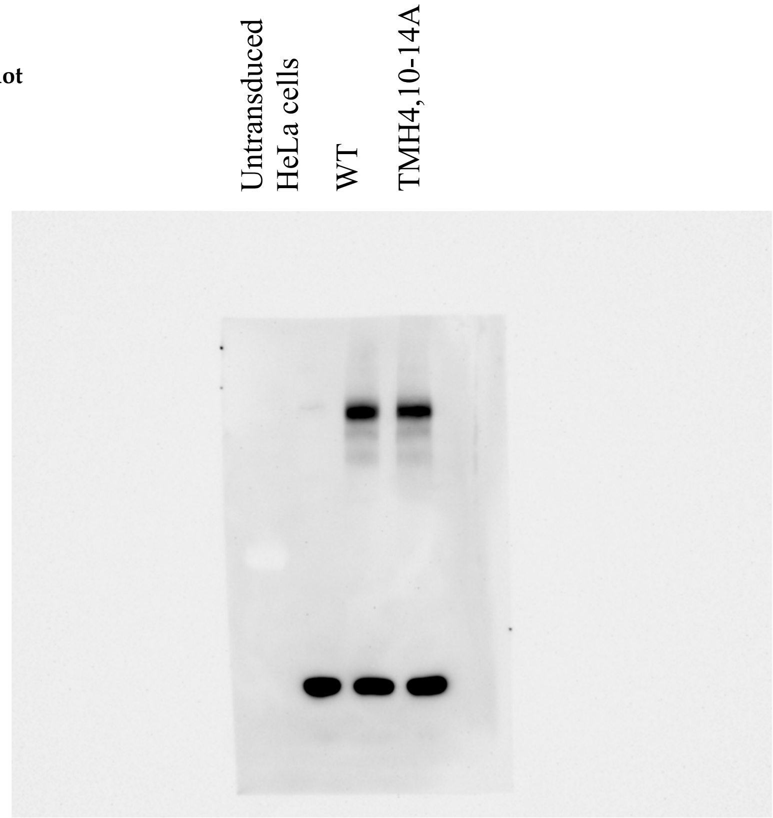

(This is the **original blot for the Figure 3E** in the main article)

Figure S5

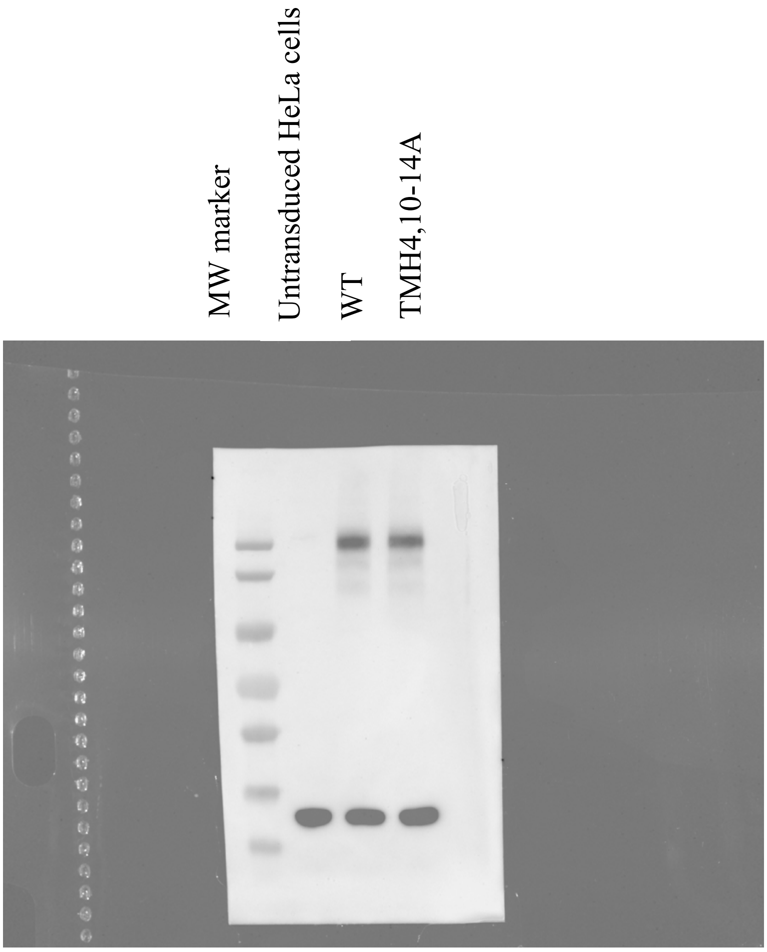

(This is the **original blot with the Molecular weight marker for the Figure 3E** in the main article. This figure was a composite of the chemiluminescence image of the blot and combined with the colorimetric figure of the molecular weight marker.)

Figure S6. Original protein-stained gel

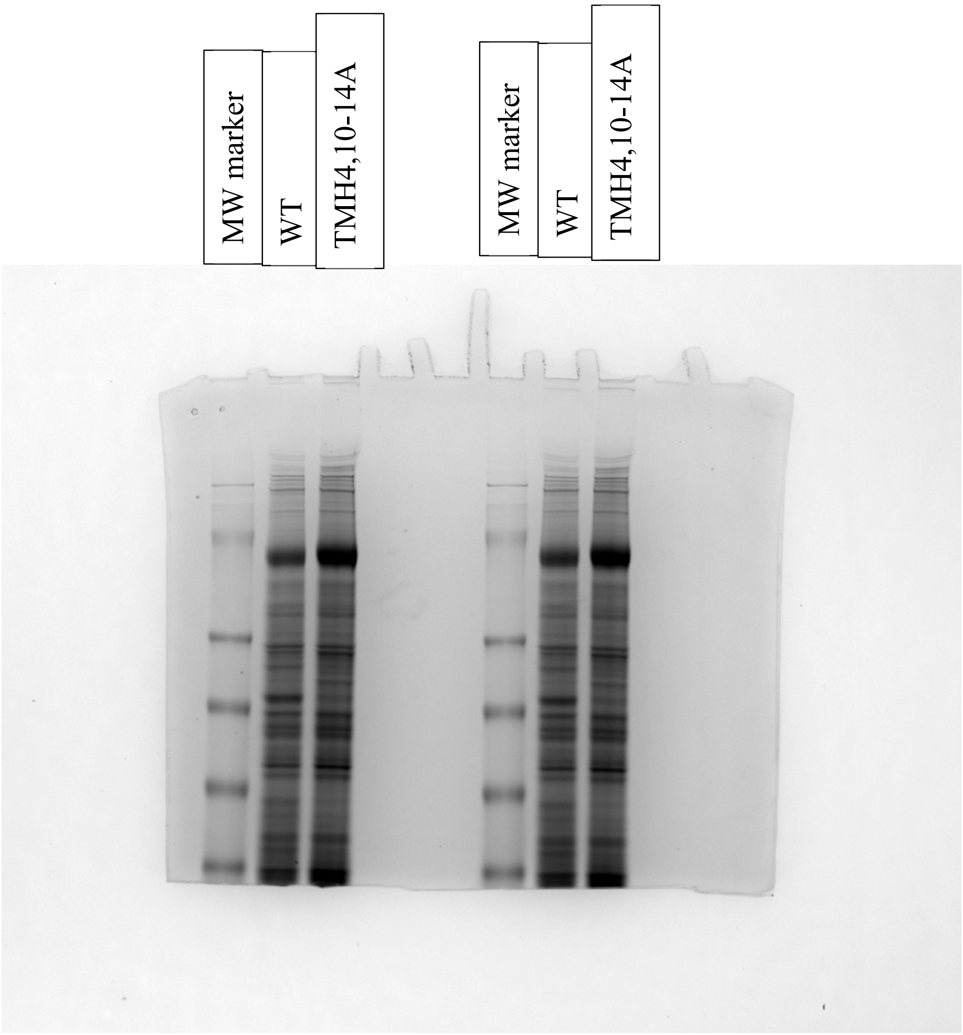

(This is the **original protein stain for the figure 6A** in the main article)
